# Telestroke management during the Hajj seasons 2023–2024: insights from SEHA Virtual Hospital in KSA

**DOI:** 10.3389/fneur.2025.1573275

**Published:** 2025-11-13

**Authors:** Ghadah Sulaiman Alsaleh, Abdulaziz Suliman A. Alhomod, Anas Khan, Saeed S. Alzahrani

**Affiliations:** 1 Global Center for Mass Gathering Medicine, Ministry of Health, Riyadh, Saudi Arabia; 2 SEHA Virtual Hospital, Ministry of Health, Riyadh, Saudi Arabia; 3 National Neuroscience Institute, King Fahad Medical City, Riyadh, Saudi Arabia

**Keywords:** telemedicine, telestroke, stroke, Hajj, mass gatherings, ischemic stroke, thrombolysis

## Abstract

**Background:**

The Hajj pilgrimage in Saudi Arabia poses significant healthcare challenges due to overcrowding and high demand. Stroke, a leading cause of morbidity and mortality, necessitates timely management, with telemedicine providing crucial remote access to specialized care.

**Objectives:**

This study aimed to evaluate the demographic and clinical characteristics of pilgrims who experienced strokes and utilized telemedicine during the Hajj seasons of 2023 and 2024, focusing on identifying changes in patient profiles and treatment patterns over time.

**Methods and materials:**

A cross-sectional study was conducted using data from SEHA Virtual Hospital (SVH) during the Hajj seasons of 2023 and 2024. The study included 459 adult stroke patients (207 in 2023 and 252 in 2024) who completed telemedicine consultations. Demographic and clinical data, including stroke types and treatment details, were retrospectively analyzed. Statistical comparisons were performed using chi-square and Mann–Whitney *U* tests, with significance set at *p* < 0.05.

**Results:**

The mean age of patients was 64.13 years in 2023 and 62.43 years in 2024. Non-Saudi patients constituted 69% in 2023, decreasing to 62% in 2024, while Saudi patients increased from 31 to 38%. Male patients rose from 57% in 2023 to 62% in 2024. Diagnoses showed significant variation: ischemic strokes increased from 46% in 2023 to 73% in 2024, while hemorrhagic strokes and other miscellaneous conditions decreased, and TIAs showed a slight increase. TPA administration remained stable at 23% in both years, and the median time to TPA administration showed no significant difference.

**Conclusion:**

The consistent application of a centralized telestroke service during the Hajj demonstrates that telemedicine is a viable and scalable strategy for delivering specialized stroke care in mass gathering settings. This model successfully managed a large, diverse patient cohort and maintained stable acute treatment rates, underscoring its potential to enhance health system resilience and equity during the most logistically challenging events.

## Introduction

Telemedicine has revolutionized healthcare delivery by enabling remote access to medical consultations, diagnostics, and treatments (1). This advancement has become especially valuable in challenging contexts such as mass gatherings, where patient volume is high and logistical barriers can impede timely care (2, 3). The annual Hajj pilgrimage in Saudi Arabia, which attracts over 2 million Muslims, presents a paramount example (4, 5). The massive influx of pilgrims creates a temporary population density that places exceptional strain on the Saudi healthcare system. A core challenge is the delivery of specialized medical care, as the limited number of on-site specialists must serve a population that temporarily rivals that of a major city, creating a patient-to-specialist ratio that is highly challenging traditional in-person healthcare delivery. This context makes telemedicine a critical tool for equitable and efficient service delivery (6, 7).

Stroke is a leading cause of death and long-term disability globally, responsible for 7.44 million deaths in 2021 (8). In Saudi Arabia, the annual incidence is approximately 29 cases per 100,000 people, as reported in a systematic review and meta-analysis (9). The physically demanding rituals of Hajj, combined with the extreme temperatures and the need for pilgrims to engage in strenuous activities, can exacerbate pre-existing conditions like hypertension or diabetes, leading to increased cardiovascular risks, including strokes (10). Stroke management, a critical and time-sensitive area of medicine, is particularly impacted during this period. Thus, timely and efficient stroke care requires innovative solutions to overcome delays caused by overcrowding and the strain on healthcare infrastructure (11).

Telemedicine emerges as a key tool, allowing specialists to provide rapid interventions remotely, thereby reducing the risk of adverse outcomes and enhancing the quality of healthcare services during this critical period. It has proven to be an effective tool in addressing these challenges, enabling real-time access to specialized care (12). Studies have highlighted that regions employing telemedicine experience reduced delays in administering critical treatments like tissue plasminogen activator (tPA), ultimately leading to improved patient outcomes (13). These advancements demonstrate the growing role of telemedicine in meeting the complex healthcare demands of mass gatherings like Hajj.

Although telemedicine has demonstrated success in improving stroke care in various settings, its use during mass gatherings such as Hajj is understudied. The Hajj pilgrimage presents distinct challenges, including the need to cater to a highly diverse and transient population, many of whom are elderly or from underserved regions. Additionally, logistical barriers such as overcrowding and limited on-site resources further complicate healthcare delivery (14, 15). Understanding how telemedicine can adapt to these conditions is crucial for optimizing stroke management during the Hajj.

This study aimed to evaluate the demographic and clinical characteristics of pilgrims who experienced strokes and utilized telemedicine during the Hajj seasons of 2023 and 2024, focusing on identifying changes in patient profiles and treatment patterns over time. The study analyzes data from SEHA Virtual Hospital (SVH), specifically exploring factors such as stroke subtypes, patient demographics, and treatment patterns, including tissue plasminogen activator (tPA) administration. This analysis will contribute to understanding the role of telemedicine in stroke management during mass gatherings like Hajj and highlight trends in patient care, which can help refine strategies for improving healthcare delivery in high-demand situations.

## Methods and materials

### Study design and participants

This cross-sectional study was conducted to evaluate the demographic and clinical characteristics of Hajj pilgrims who experienced strokes and utilized telemedicine during the Hajj seasons in Saudi Arabia. The study population was exclusively composed of Hajj pilgrims. Pilgrim status was actively verified at the point of clinical presentation and systematically documented in the medical record by the treating healthcare team as part of the standardized intake procedure during the Hajj season. The study periods were from June 26 to July 1, 2023, and from June 14 to June 19, 2024. It was conducted at SEHA Virtual Hospital (SVH), established in 2022 by the Saudi Ministry of Health (MOH) in Riyadh as the world's largest and the Middle East's first fully virtual hospital. SVH functions as a central hub, receiving real-time telemedicine consultations from community and regional hospitals across the Kingdom and providing immediate, specialist-led treatment recommendations. The establishment of SVH is a flagship initiative of the Saudi Digital Health Strategy, a national framework designed to transform healthcare delivery through digitalization. A key objective of this strategy is to enhance access to specialized medical expertise across the vast geography of the Kingdom and during high-demand events, thereby improving healthcare quality and efficiency (16). SVH provides remote healthcare services to patients across Saudi Arabia, leveraging real-time consultations, advanced monitoring systems, and telemedicine applications. During the Hajj season, SVH serves an increased patient load, addressing the heightened demand for specialized medical services, including stroke management. The SVH telemedicine system uses advanced technology, allowing real-time consultations with specialized stroke teams across the Kingdom. Each pilgrim who experienced a stroke had their clinical information, including diagnostic imaging, vital signs, and laboratory results, shared via secure platforms during telemedicine sessions.

### SEHA Virtual Hospital telestroke service and network

The SEHA Virtual Hospital (SVH) telestroke service operates as a hub-and-spoke model to provide acute stroke expertise to facilities across the Kingdom. While this study focused exclusively on Hajj pilgrims, the telestroke system is a national service available year-round and accessible to non-pilgrim patients across the network of 57+ hospitals connected to SVH. During the Hajj season, the network is strategically reinforced to serve the holy cities of Makkah and Madinah, where the vast majority of pilgrims are concentrated. The spoke hospitals most affected by Hajj-related patient surges are those located in the central Hajj areas of Makkah (proximate to the Grand Mosque, Mina, and Arafat) and in Madinah (near the Prophet's Mosque).

Teleconsultations are hospital-initiated, accessed from emergency departments of hospitals participating in the Hajj telestroke network. Patients themselves do not connect directly; the consultation is initiated by the hospital team when a suspected stroke is identified. The process is triggered when a suspected stroke patient presents to the emergency department of a participating spoke hospital. The local team activates a “telestroke code” by calling a dedicated hotline to the SVH hub. Hospitals are equipped with dedicated telemedicine workstations and carts that include high-resolution video cameras, screens, and secure connections to the SEHA Virtual Hospital (SVH) hub. This allows remote neurologists to directly observe the patient and communicate with the local clinical team. Smartphones or personal devices are not used.

Upon connection, a remote stroke neurologist performs a real-time, directed neurological examination of the patient via a secure, high-definition videoconferencing platform. All consultations occur through a secure, encrypted platform integrated into the national digital health infrastructure. Patient identifiers and imaging data are transmitted via encrypted channels compliant with Ministry of Health (MOH) data governance policies, and access is restricted to authorized healthcare providers only. The neurologist concurrently reviews the patient's neuroimaging, including non-contrast CT and CTA scans, via direct access to the Picture Archiving and Communication System (PACS). The diagnosis and treatment decision are based on the integration of this real-time clinical information, which includes the patient's history, neurological examination findings (e.g., NIHSS), laboratory results (e.g., coagulation profile, blood glucose), and neuroimaging, all transmitted securely from the spoke hospital's electronic medical record and PACS systems to the SVH platform. Based on this comprehensive assessment and guideline-directed criteria, the SVH neurologist advises on eligibility for intravenous thrombolysis. Consent for thrombolysis (rt-PA) is obtained at the spoke hospital by the treating emergency physician or designated staff, in line with Saudi Ministry of Health policies. The SVH neurologist provides the treatment recommendation, outlines the risks and benefits to facilitate the informed consent process, and guides the local team.

This integrated approach enables the remote specialist to provide definitive treatment recommendations. For acute ischemic stroke patients, the SVH neurologist confirms the diagnosis, assesses eligibility, and authorizes the administration of intravenous recombinant tissue plasminogen activator (rt-PA) at the presenting hospital. The SVH team guides the local clinicians through dosing, monitors the patient during infusion via the video/audio link, and manages any complications. This process avoids the delays associated with transferring the patient to a tertiary center, significantly reducing door-to-needle time. For patients requiring mechanical thrombectomy, the SVH specialist coordinates immediate transfer to a comprehensive stroke center. By enabling the remote initiation of thrombolysis, the virtual hospital critically expands access to reperfusion therapy and improves equity of stroke care, especially during high-demand events like the Hajj.

To address the multicultural and multilingual nature of the Hajj pilgrimage, several systemic measures are in place to mitigate communication barriers and ensure effective telemedicine delivery. During the Hajj season, hospitals are supported by on-site volunteer interpreters, and many official Hajj medical missions provide their own interpreters for their pilgrims. Furthermore, the Saudi Ministry of Health's 937 Emergency Call Center offers centralized telephonic interpretation support in major languages, including Urdu, French, Swahili, Spanish, Indonesian, and Farsi. If a language barrier is encountered during a teleconsultation, the SVH neurologist can connect with the 937 center to facilitate real-time interpretation. Additionally, local healthcare providers at spoke hospitals receive annual training on activating the telestroke system and collaborating effectively with the remote specialist team, ensuring a standardized and proficient approach to telemedicine consultations.

**Figure 1:**
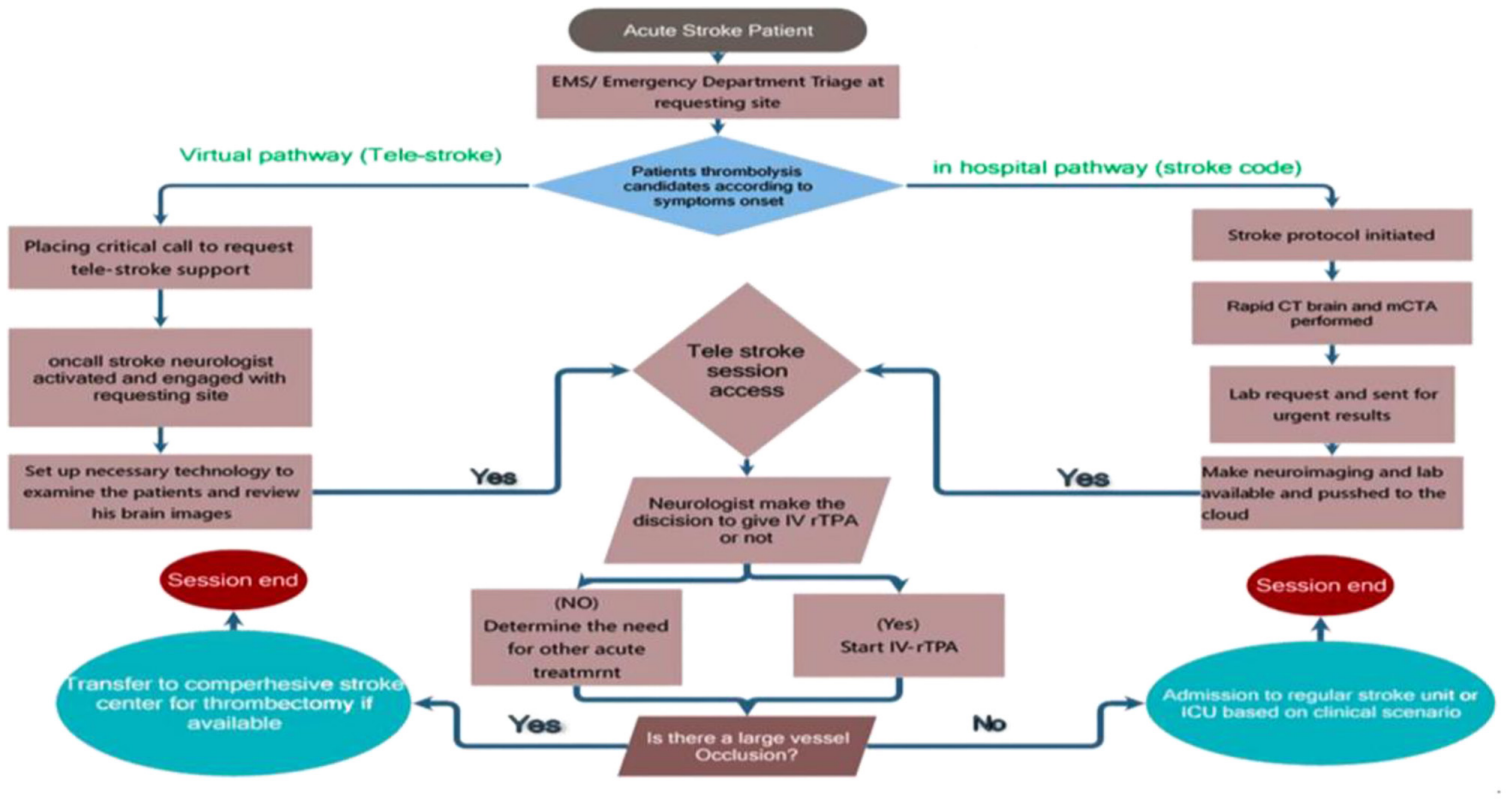
Workflow of the SEHA Virtual Hospital telestroke service during the Hajj season.

**Figure 2:**
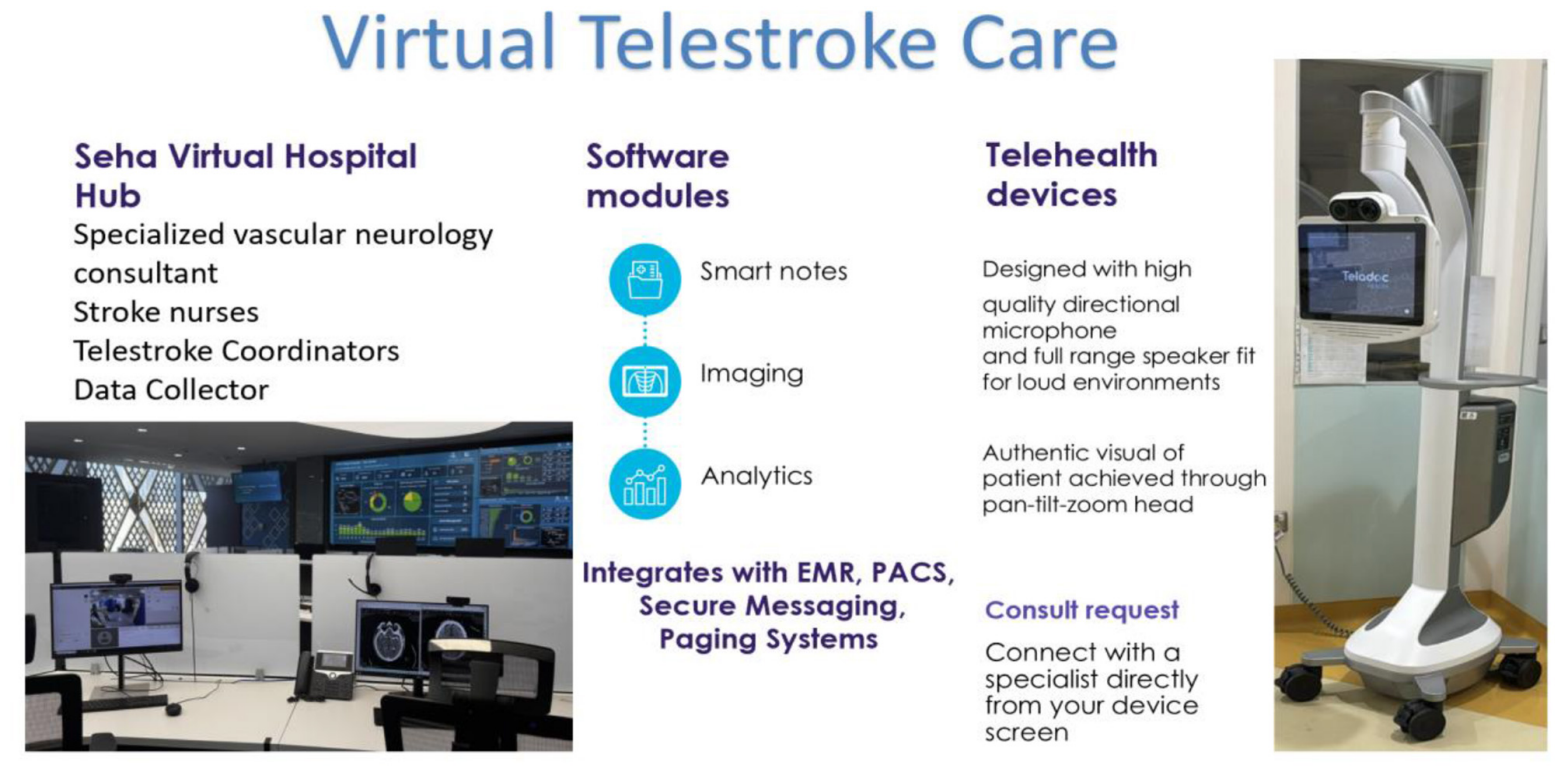
Virtual telestroke care. Photograph by Saeed Saleh Alzahrani (SEHA Virtual Hospital, October 2023); used with permission.

A schematic of this workflow is provided in Figure 1 and a visual overview of the telemedicine ecosystem and its components is provided in Figure 2.

The catchment area of this service during the Hajj is unique, extending to the millions of pilgrims who arrive annually from over 180 countries. This international composition of the pilgrim population explains the high proportion of non-Saudi patients (62%−69%) characterized in the present study cohort, which stands in contrast to the demographic profile typically served by the Saudi healthcare system outside of the Hajj period.

### Study period and program enhancements

The analysis was focused on two consecutive Hajj seasons, 2023 and 2024. This approach was selected due to the unique nature of the Hajj as a mass gathering event, which is distinct from routine healthcare delivery. Each season is characterized as a discrete, highly standardized period with a large and diverse patient influx, allowing for a direct comparison under consistently challenging conditions, including overcrowding, extreme heat, and significant logistical constraints.

The 6-day study period for each Hajj season was selected based on the unique operational framework of the Hajj healthcare system. The spoke hospitals included in this analysis, located in the central holy sites of Makkah (Mina, Arafat, and Muzdalifah) are seasonal facilities. These hospitals are established and operate exclusively during the six official days of the Hajj pilgrimage to serve the millions of pilgrims gathered in these specific locations. Outside of this period, these facilities are closed. Therefore, this defined 6-day window is not a sampling period but rather captures the entire operational lifespan of the telestroke service within the core Hajj grounds. This condensed, high-intensity timeframe also underscores the significant logistical achievement of implementing and operating a complex telestroke system with annually rotating clinical staff, highlighting the system's robustness under extreme conditions.

It is acknowledged that a 2-year comparison does not establish a long-term temporal trend. The objective was not to project a trajectory, but to identify early signals of change in patient profiles and clinical management following the introduction and subsequent scale-up of the SVH telestroke program. Importantly, between the 2023 and 2024 Hajj seasons, specific structural enhancements were implemented to strengthen the service:

- Network coverage was expanded to include additional hospitals in Makkah and Madinah.
- Staffing capacity was increased, with a greater number of on-call stroke consultants and coordinators made available during peak hours.
- Diagnostic triage was improved through the enhanced use of standardized stroke protocols and closer coordination with emergency departments.

These programmatic adjustments are considered essential context for the interpretation of the findings between the two study periods.

### Sample size

A total of 459 stroke patients (207 in 2023 and 252 in 2024) were included in this study. The sample size was not calculated *a priori* but rather included all eligible stroke patients referred to SVH during the study period.

### Inclusion and exclusion criteria

The inclusion criteria for this study encompassed adult patients aged 18 years and older diagnosed with stroke conditions, including ischemic, hemorrhagic, transient ischemic attack (TIA), or other related conditions. Eligible participants were those referred to SEHA Virtual Hospital (SVH) for telemedicine consultations during the Hajj seasons of 2023 or 2024 and who had completed at least one telemedicine session with available clinical data. Conversely, exclusion criteria were applied to pediatric patients under the age of 18, individuals with incomplete medical records or missing critical clinical variables, and those not evaluated during the specified study periods.

### Procedure and data collection

This study relied on a retrospective review of electronic medical records from SVH. Data collection involved retrieving patient demographics (nationality and gender), clinical characteristics [stroke type (ischemic, hemorrhagic, TIA, or other), administration of tissue plasminogen activator (TPA), and time to TPA]. The data collection and review process adhered to predefined protocols, ensuring consistency and reliability of the extracted variables. No experimental manipulations or additional procedures were performed beyond standard clinical protocols for telemedicine consultations, which were conducted through video consultations provided by a multidisciplinary team. This team includes emergency physicians, neurologists, and other relevant specialists, depending on the patient's condition.

### Ethical considerations

This study was conducted in accordance with the ethical principles outlined in the Declaration of Helsinki, which ensures the protection of the rights, safety, and wellbeing of participants involved in clinical research. Ethical approval was granted by the Research Ethics Committee at King Fahad Medical City (KFMC), with the approval documented under IRB log Number: 23-697E. For this study, verbal consent was obtained from all participants prior to their telemedicine consultation. All data were fully anonymized prior to analysis. The confidentiality of participants' personal information was maintained, and all data were anonymized to protect privacy.

### Statistical analysis

Statistical analysis was done by SPSS version 28 (IBM Co., Armonk, NY, USA). The selection of statistical tests was based on the nature and distribution of the data. Categorical variables, including nationality, gender, diagnosis type, and TPA administration, were presented as frequencies and percentages. Differences in the distribution of these variables between the 2023 and 2024 cohorts were assessed using the Chi-Square test. For the continuous variable “time to TPA administration,” which was not normally distributed, data were reported as median and interquartile range (IQR), and the Mann–Whitney *U* test was used for comparison between the 2 years. No replacement or imputation were done for the missing data. Missing data were only one data point for age, one for nationality and one for time to TPA. A *p* value <0.05 was considered statistically significant.

## Results

The demographic and clinical characteristics of stroke patients utilizing telemedicine during the Hajj seasons of 2023 and 2024 in Saudi Arabia are presented in Table 1. The mean age of patients was 64.13 years (SD = 10.59) in 2023, slightly decreasing to 62.43 years (SD = 12.61) in 2024, with no statistically significant difference between the 2 years (*p* = 0.118). In 2023, 69% of the patients were non-Saudi, which decreased to 62% in 2024, while Saudi nationals increased from 31% in 2023 to 38% in 2024 (*p* = 0.098). Regarding gender, male patients constituted 57% of the cohort in 2023, increasing to 62% in 2024, whereas the proportion of female patients decreased from 43 to 38% during the same period (*p* = 0.287). The distribution of diagnoses showed significant variation between the 2 years (*p* < 0.001): Hemorrhagic strokes constituted 5% of cases in 2023, decreasing to 4% in 2024, while ischemic strokes demonstrated a substantial increase, rising from 46% in 2023 to 73% in 2024. The incidence of transient ischemic attacks (TIAs) exhibited a modest increase, from 3% in 2023 to 5% in 2024. Conversely, the proportion of other diagnoses, including stroke mimics such as hypoglycemia, migraines, brain tumors, and other conditions, declined markedly from 45% in 2023 to 18% in 2024. The administration of tissue plasminogen activator (TPA) remained stable, with 23% of patients receiving TPA in both years (*p* = 0.982). The median time to TPA administration was 77.5 min [interquartile range (IQR): 63–107.75] in 2023 and 85 min (IQR: 66–94) in 2024, with no statistically significant difference observed (*p* = 0.897).

**Table 1:** Demographic and clinical characteristics of stroke patients utilizing telemedicine during the Hajj seasons of 2023 and 2024 in Saudi Arabia.

	**2023**	**2024**	***p*-Value**
	***N*** = **207**	***N*** = **252**	
**Age**			
Mean (SD)	64.13 (10.59)	62.43 (12.61)	0.118
	***N*** **(%)**	***N*** **(%)**	
**Nationality**			
Non-Saudi	142 (69%)	155 (62%)	0.098
Saudi	64 (31%)	97 (38%)	
**Gender**			
Female	89 (43%)	96 (38%)	0.287
Male	118 (57%)	156 (62%)	
**Diagnosis**			
Hemorrhagic	11 (5%)	9 (4%)	<0.001
Ischemic	95 (46%)	184 (73%)	
TIA	7 (3%)	13 (5%)	
Other	94 (45%)	46 (18%)	
**TPA given**			
No	160 (77%)	195 (77%)	0.982
Yes	47 (23%)	57 (23%)	
**Time to TPA (minutes)**	***N*** = **46**	***N*** = **57**	
Median (Q1, Q3)	77.5 (63, 107.75)	85 (66, 94)	0.897

## Discussion

The aim of this study was to evaluate the demographic and clinical characteristics of pilgrims who experienced strokes and utilized telemedicine during the Hajj seasons of 2023 and 2024, with a particular focus on identifying changes in patient profiles and treatment patterns over time. By analyzing data from SEHA Virtual Hospital (SVH), the findings reveal trends in patient demographics, stroke subtypes, and treatment metrics, such as tissue plasminogen activator (TPA) administration and timing.

In 2023, 69% of stroke patients using telemedicine were non-Saudi, decreasing to 62% in 2024. Conversely, Saudi nationals increased from 31 to 38%. While this change was not statistically significant (*p* = 0.098), it suggests a shift in access or utilization trends among the local population. The observed increase in telemedicine adoption among Saudi nationals aligns with findings from Abd El Mawgod et al. (17) who reported that despite a low level of awareness of telemedicine among the Saudi general population, there is a positive perception and high willingness to adopt this technology. The study highlighted barriers, such as concerns about diagnostic reliability, resistance from physicians, and patient hesitancy, which may also influence the gradual increase in local uptake (17). Targeted public health initiatives and increased familiarity with telemedicine could explain the rising utilization among Saudi nationals observed in your study, particularly during high-demand periods like Hajj. This shift may be driven by government-led initiatives, including the Saudi Digital Health Strategy, which aims to enhance telemedicine accessibility across the kingdom. For instance, recent reports suggest that the Kingdom's telehealth market has experienced substantial growth, driven by rising demand for remote healthcare services, public-private partnerships, and the regulatory frameworks. These likely contributed to bridging gaps in telemedicine adoption, improving access for Saudi citizens during events like the Hajj (18). However, the significant proportion of non-Saudi patients underscores the importance of continuing to provide equitable care to international pilgrims. This includes ensuring multilingual support and culturally tailored services, which are crucial for addressing the diverse needs of Hajj participants.

On the other hand, our result contrasts with findings from Talmesany et al. (19) who reported a generally low level of familiarity with telemedicine services among the population in the Al-Baha region in Saudi Arabia, with only 54.9% of participants having used telemedicine. The study highlighted major barriers such as concerns about privacy, quality of care, and limited availability, which may inhibit broader adoption of telemedicine services (19). These findings suggest regional and contextual differences in telemedicine utilization across Saudi Arabia. While the observed increase in Saudi patients utilizing telemedicine during the Hajj may stem from targeted public health campaigns and immediate healthcare demands, Talmesany et al.'s (19) findings emphasize the need to address systemic barriers in other regions. The cultural and infrastructural diversity across the kingdom likely accounts for the variance in telemedicine utilization. Mass gatherings like Hajj provide a unique context where accelerated adoption is observed due to the critical need for accessible healthcare.

The observation that male patients constituted 57% of the cohort in 2023, increasing to 62% in 2024, whereas the proportion of female patients decreased from 43 to 38% during the same period, aligns with broader trends in gender disparities in stroke care. Globally, these differences in healthcare access and utilization often stem from cultural, societal, and systemic factors. Research by Khosravi et al. (20) identified that Middle Eastern women face barriers to healthcare utilization stemming from limited education and awareness, emotional barriers such as embarrassment, and restrictive cultural norms. The study suggested that technologies like telemedicine, particularly mHealth, could bolster women's access to healthcare services in the region by addressing some of these challenges (20). This perspective underscores the need for targeted campaigns and educational interventions to increase telemedicine adoption among women in Saudi Arabia and beyond. Similarly, Habib et al. (21) confirmed significant gender disparities in access to healthcare services in Saudi Arabia. Their study highlighted systemic barriers such as logistical challenges and cultural norms that disproportionately hinder women's access compared to men (21). These findings suggest that while initiatives like the Saudi Digital Health Strategy aim to enhance telemedicine accessibility, gender-specific considerations must be integrated to ensure equitable adoption across all population segments. Further evidence from Albaghdadi and Al Daajani revealed that women were more likely to report lower satisfaction with telemedicine consultations compared to men, emphasizing the need for gender-sensitive approaches to telemedicine delivery (22). Addressing factors such as privacy concerns, communication styles, and quality of care could improve women's experiences and outcomes with telemedicine. Finally, a report from the Middle East Policy Council supports the broader narrative of persistent gender inequality in healthcare access across the MENA region. Cultural norms, financial constraints, and education gaps exacerbate these disparities, hindering women's ability to benefit fully from telemedicine and other healthcare innovations (23). These insights collectively highlight the importance of addressing gender-specific barriers to telemedicine adoption, particularly during mass gatherings like Hajj, where timely access to care is critical. Bridging these gaps requires concerted efforts in policy, education, and technological innovation tailored to the needs of women.

In our results, the predominance of ischemic strokes aligns with global trends, as ischemic strokes account for approximately 87% of all strokes worldwide (24). The significant reduction in “other” diagnoses, which include stroke mimickers such as hypoglycemia, migraines, and brain tumors, may reflect improved diagnostic accuracy facilitated by telemedicine. Additionally, accurate triage protocols and targeted training of healthcare staff at spoke hospitals play a pivotal role in reducing the occurrence of stroke mimickers and other non-stroke diagnoses (25). The increase in ischemic strokes observed in our study is consistent with findings in another telemedicine-related research. A study by Evans et al. (26) highlights the importance of telemedicine in ischemic stroke care, demonstrating that it enables timely treatment through remote assessments, particularly for thrombolysis delivery. This aligns with our result that the focus on ischemic strokes increased, potentially due to improved diagnostic accuracy facilitated by telemedicine.

Moreover, our findings regarding the slight increase in transient ischemic attacks (TIAs) and the decrease in “other” diagnoses may reflect a broader global trend, where telemedicine enhances clinical decision-making, leading to more accurate diagnoses (27). The use of telemedicine in stroke care has been associated with improved diagnostic outcomes, which could explain the marked reduction in unspecified diagnoses in our study.

In our study, the proportion of patients receiving tissue plasminogen activator (TPA) remained stable at 23% across both years (*p* = 0.982). This finding aligns with global trends in TPA administration among eligible stroke patients, though rates can vary depending on healthcare systems and regional protocols. While studies have reported variable TPA administration rates, telemedicine-supported stroke care has demonstrated increased TPA usage due to improvements in door-to-needle times (28). For example, telemedicine-based stroke care networks, such as hub-and-spoke models, have been associated with a higher proportion of patients receiving timely thrombolytic treatment by streamlining decision-making and reducing transport delays (29). Moreover, recent studies have shown that telemedicine can enhance stroke treatment by facilitating faster diagnosis and coordination between healthcare teams, thus increasing the likelihood of administering thrombolysis within the critical treatment window (30).

The stability in TPA usage in our study suggests consistent adherence to established treatment protocols at SVH. However, there remains potential for improvement. Efforts to optimize stroke care workflows—especially integrating telemedicine more deeply into the clinical process—could further enhance the proportion of eligible patients receiving thrombolytic therapy. Specifically, reducing delays in decision-making and transport could increase the number of patients receiving timely treatment, mirroring findings from other studies that emphasize the role of telemedicine in expediting stroke care.

The median time to TPA administration was 77.5 min in 2023 and 85 min in 2024, with no significant difference observed (*p* = 0.897). However, the lack of significant improvement in our study may reflect logistical challenges unique to the Hajj setting, such as crowd density and transportation delays, which can complicate timely patient transfer. Despite the potential benefits of telemedicine, external factors may still delay the treatment timeline. Nevertheless, telemedicine systems have been associated with reduced door-to-needle times in acute stroke care (31). For example, a study by Blech et al. (32) found that Telestroke network participation may be associated with improved acute stroke care metrics over time, with the analysis illustrating improved last-known normal to needle and door-to-needle times among participating spoke sites (32). On another point, a study by Loggini et al. (33) found that in-person evaluation provided faster door-to-needle time compared to telemedicine. This trend reversed when a midlevel provider facilitated telemedicine. The faster door-to-needle time did not translate into increased safety or better short-term outcome (33). When contextualized against international benchmarks such as the American Heart Association's goal for a time to TPA of ≤ 60 min, our median times highlight the persistent challenge of achieving gold-standard metrics in mass gathering settings, despite the facilitating role of telemedicine (34). Streamlining communication between remote facilities and SVH specialists is crucial to minimize delays in TPA administration. Additionally, investments in prehospital stroke care, such as mobile stroke units, could complement telemedicine efforts and further improve outcomes. Incorporating such technologies into the Hajj setting could enhance patient outcomes during peak periods.

## Strengths and limitations

This study's strengths lie in its novel focus on telemedicine for stroke management during the unique and high-demand context of the Hajj pilgrimage. The use of data from SEHA Virtual Hospital, the world's largest virtual hospital, ensures a robust and comprehensive dataset, capturing real-world clinical practices across two consecutive Hajj seasons. The cross-sectional design enabled the evaluation of demographic and clinical trends, highlighting changes in patient profiles and treatment patterns. Additionally, the study provides insights into the application of telemedicine in a resource-intense and logistically challenging setting, contributing valuable knowledge to a growing field. However, limitations include the inability to establish causation due to the cross-sectional nature of the study, potential selection bias since data were derived exclusively from a single virtual hospital, and the lack of detailed information on patient outcomes or satisfaction. A key limitation inherent to the Hajj context is the absence of data on critical clinical outcomes such as the modified Rankin Scale (mRS) at 3 months or mortality, as the transient pilgrim population returns to their home countries shortly after the acute event, making longitudinal follow-up unfeasible. Furthermore, the absence of a comparative baseline from off-season periods makes it difficult to precisely quantify the specific impact and added strain of the Hajj season. This comparison would have provided valuable context to better understand the unique challenges and adaptations required during this event. Future research should consider longitudinal designs, incorporate off-season data for comparison, and utilize multi-center data to validate and expand on these results.

## Conclusion

This study confirms the critical role and operational feasibility of a centralized telestroke service in managing acute stroke during the Hajj pilgrimage. The SEHA Virtual Hospital model proved effective in serving a large, international patient cohort and maintained consistent rates of thrombolysis across two seasons. Beyond demonstrating feasibility, the evolving patient profile, marked by a significant increase in diagnosed ischemic strokes and a growing proportion of Saudi nationals, suggests that the system is maturing, with improved diagnostic accuracy and increasing integration into the domestic healthcare ecosystem. While logistical challenges inherent to the Hajj setting persist, this telemedicine framework provides a robust and adaptable blueprint for delivering equitable, specialist-led care during mass gatherings. The insights gained are invaluable for informing the design of emergency healthcare services for other large-scale global events.

## Future recommendations

To build upon these findings, future studies should adopt longitudinal designs to assess patient outcomes, satisfaction, and long-term impacts of telemedicine interventions. The application of multivariable regression models in future studies with larger, more comprehensive datasets would be valuable to identify independent predictors of key outcomes, such as tPA administration, while controlling for potential confounders. Additionally, establishing international collaborative frameworks or digital registries to enable the tracking of long-term clinical outcomes (e.g., mRS, mortality) for pilgrims after they return to their home countries is a critical next step for evaluating the sustained effectiveness of telemedicine interventions. Expanding the dataset to include multi-center collaborations could provide a more comprehensive understanding of telemedicine's efficacy in diverse healthcare settings. Furthermore, incorporating qualitative research to explore patient and provider experiences could identify barriers and facilitators to telemedicine adoption. Finally, targeted efforts to streamline TPA administration times and address disparities in care delivery during high-demand periods, such as the Hajj, will be essential in optimizing telemedicine for stroke management and improving overall healthcare outcomes.

## Data Availability

The original contributions presented in the study are included in the article/Supplementary material, further inquiries can be directed to the corresponding author.
